# Effect of Particle Morphology on Stiffness, Strength and Volumetric Behavior of Rounded and Angular Natural Sand

**DOI:** 10.3390/ma14113023

**Published:** 2021-06-02

**Authors:** Aashish Sharma, Alexia R. Leib-Day, Mohmad Mohsin Thakur, Dayakar Penumadu

**Affiliations:** 1 GEOServices LLC, Cleveland, TN 37312, USA; asharma@geoservicesllc.com; 2 Dan Brown and Associates, PC, Knoxville, TN 37919, USA; ali@dba.world; 3 Department of Civil and Environmental Engineering, Tickle College of Engineering, The University of Tennessee, Knoxville, TN 37996, USA; mthakur1@vols.utk.edu; 4 Fred N. Peebles Professor and JIAM Chair of Excellence, Department of Civil and Environmental Engineering, Tickle College of Engineering, The University of Tennessee, Knoxville, TN 37996, USA

**Keywords:** sand, particle shape, particle crushing, engineering properties

## Abstract

Stress–strain and volume change behavior for clean sands which have distinct particle shape (rounded and angular) with very similar chemical (mineralogical) composition, size, and texture in one-dimensional (1D) compression and drained triaxial compression are presented. The effect of particle morphology on the crushing behavior in one-dimensional loading is explored using laser light diffraction technique which is suitable for particle crushing because of its high resolution and small specimen volume capability. Particle size distribution in both volume/mass and number distributions are considered for improved understanding associated with the process of comminution. Number distributions present a clearer picture of particle crushing. It is argued that particle crushing in granular assemblies initiates in larger particles, rather than in smaller particle. It was found that rounded sand specimens showed greater crushing than angular sand specimens with higher uniformity coefficient. In 1D compression, loose specimens compress approximately 10% more than dense specimens irrespective of particle shape. Densification of angular sand results in improvement in stiffness (approximately 40%) and is comparable to that of loose rounded sand. In general, density has a greater influence on the behavior of granular materials than particle morphology. The effect of particle shape was found to be greater in loose specimens than in dense specimens. The effect of grain shape on critical state friction angle is also quantified.

## 1. Introduction

Understanding the depositional characteristics of granular materials and their response to external load is important in many geotechnical applications. Applications may range from foundation engineering [1], projectile penetration, Refs. [2,3,4] to improving loose deposits by reinforcements [5], and to creating composite backfills such as sand-rubber mixtures [6,7].

The angle of shearing resistance is an important measure of the shear strength of granular materials. The friction angle of coarse-grained soils depends on several factors, chief among them are: the size of the particles [8], the texture or surface roughness of the grains [9,10], mineralogy of the grains [8] and shape of the particles [8,10,11,12,13,14,15,16,17,18]. Studies have also shown the influence of particle angularity on the steady state friction angle at large strain [8,19,20]. In the past the influence of particle shape on the stress–strain behavior has been evaluated either on datasets of natural and artificial materials [15,21], on material of same mineralogy but prepared in laboratory by crushing [8], on materials with different mineralogy [13], on materials with very different particle size [22], and on material prepared by mixing different fractions of rounded and angular grains [16]. Attempts have also been made to investigate the role of roughness on peak friction angle and dilatancy angle [23]; however, the effects of roughness and angularity were not decoupled. These studies have generally concluded index densities (*e*_min_, *e*_max_) increase with increasing angularity and for a given particle size rounded grains packs more densely than angular grains. Additionally, the peak friction angle increases with increasing grain angularity.

Past studies on the effect of particle morphology on the response of granular materials has addressed specific issues: (a) effect of particle shape on the peak friction angle [8,15], (b) effect of particle shape on packing and initial state [8,13,24,25], (c) effect of particle shape on dilatancy [21,22], (d) effect of angularity on the steady state friction angle [8,19,20,24], and (e) effect of roughness on the peak friction angle and dilatancy angle [23]. Recently, [24] complied a database of 25 natural sands and to investigate the role of surface roughness, based on quantitative measurements, on packing and critical state parameters. They found that roughness increased with angularity, and it was difficult to decouple the effects of angularity and roughness on the response of granular materials.

In addition, grain crushing become very important in applications that involve very high stresses, such as those present at the tip of piles during pile driving, underneath a high dam, or near the tip of penetrating projectiles. At locations of high stresses, grain fracture and fragmentation will contribute to the plastic deformation in addition to slippage and reorientation of the particles. Particle fracture will also change the gradation [26,27] and shape of the particles [27], which in turn will influence the strength and volumetric response of the crushed mass. It is, therefore, important to determine the evolution of particle shape and size during particle crushing.

Comminution is quantified using particle size distribution (PSD) curves, either by comparing the changes in the mass of the material finer than a chosen particle size [28,29,30], or based on the shift in the particle size distribution curves [31,32,33]. The PSD curves for soil specimens with particle sizes ranging from clay sized to sand sized is generally determined by sieve analysis for the coarse fraction and by hydrometer analysis for the fine fraction passing No. 200 sieve. A consistent definition of particle size can be used for a wide range of particle sizes using the laser diffraction technique. It has been shown to produce similar grain size distribution as sieve analysis and image analysis [34]. It is an especially well-suited method for characterizing comminution [35,36,37] as the technique can be used for a wide range of particle sizes, including sub-micrometer particle size [38,39].

Size distribution in geotechnical engineering is generally determined using sieve analysis even when analyzing comminution [28,32,40,41], in which considerable fines smaller than the No. 200 sieve are produced. There are two issues related to characterizing comminution using sieve analysis, one is that it becomes increasingly difficult to characterize fines that are smaller than No. 200 sieve. The other is that in mass and volume-based distributions, a few larger particles can bias the distribution towards larger particle sizes. This is more pronounced when the specimen volume is small, as in the case when analyzing comminution in dynamic impact tests in Split Hopkins Pressure Bar tests or analyzing comminution of material at the tip of projectiles in laboratory projectile penetration tests. A number distribution, even with the assumption of spherical particles can provide better visualization of fines creation especially at the comminution limit [37,42]. In the last decade image analysis and laser diffraction have been increasingly used to quantify comminution of granular materials [24,26,27,35,37,43]. These different techniques have also been combined to better characterize shape and size of crushed specimens [37].

This paper presents the role of particle shape on the behavior of granular materials via results of experiments performed on two unground sands with the same mineralogy, similar surface roughness and particle size distribution. The minerology is verified using X-ray diffraction and roughness measured using confocal microscope. Results of tests on a third sand with similar mineralogy, and surface roughness but slightly different particle size distribution are also presented. This provides an opportunity to assess the importance of grain shape on the response of granular specimens to external loads. The effect of particle morphology on the initial state of granular materials is discussed via index properties. One-dimensional (1D) compression tests, isotropic compression tests, and triaxial test results are presented to discuss the effect of density and angularity on the volumetric response and stress–strain behavior. Additionally, insights into the effect of particle morphology on grain crushing in granular material subjected to high compressive stresses are provided by analyzing 1D compression test specimens at the end of loading with laser diffraction. Particle crushing is quantified in terms of volume and number distributions. The change in particle shape after crushing is presented using a simple shape parameter derived from 2D images of particles. Based on these observations a probable process leading to comminution in granular material is also presented.

## 2. Materials

**Figure 1:**
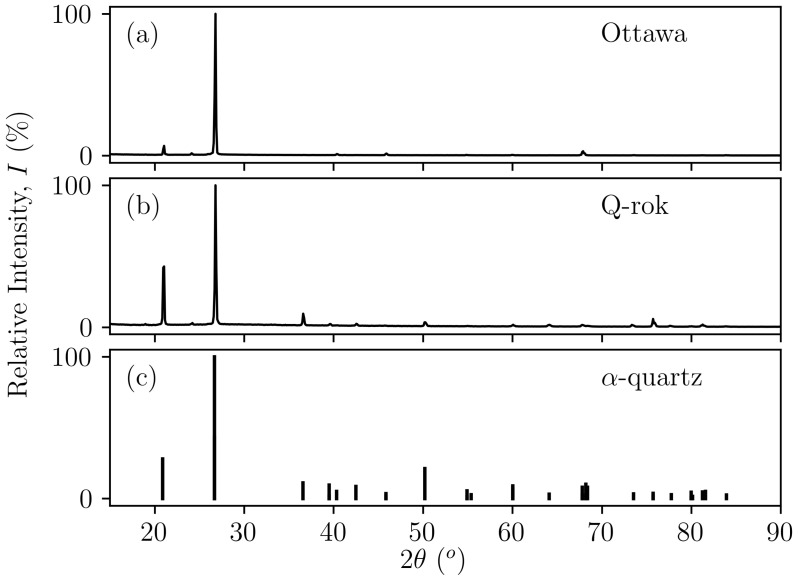
X-ray powder diffraction patterns: (**a**) Ottawa sand and (**b**) Q-Rok compared with diffraction peaks of (**c**) α-quartz.

Three commercially available sands, 20–40 Ottawa sand, Q-Rok and Euroquarz Siligran 0.125–0.71 were chosen to study the effect of grain morphology on grain crushing and stiffness in 1D compression, and strength and volumetric response in triaxial stress state. Ottawa sand grains were sub-rounded in shape while those for Q-Rok and Siligran were angular and sub-angular. Median roundness values computed from two-dimensional gray scale optical images of the 30 particles retained on US No. 30 sieve were 0.73, 0.49, 0.66 for Ottawa sand, Q-Rok and Siligran respectively. The median sphericity computed as the ratio of the width to length of the grains were 0.84, 0.76 and 0.82, respectively. These shape factors were determined from computational geometry [44] and are comparable to the shape chart in [45]. The index densities of the Siligran were very similar to the 20–40 Ottawa sand. The mineralogy of the three sands are very similar to quartz constituting more than 99.5% of the grains. X-ray powder diffraction patterns for Ottawa sand, and Q-Rok along with indexed peaks for α-quartz are shown in Figure 1. The specimens for powder diffraction were prepared by pouring the sand into powder sample holder, and gently pressing and smoothing the top to create a planar surface for diffraction. Only the peaks for those crystallographic planes that were suitably oriented are seen in the diffraction patterns of the two sands. The results indicate that both sands are mineralogically identical albeit with small differences in orientation of the crystallographic planes when deposited in air. This should have no consequence for the triaxial tests as the tests were performed at low confining stresses and particle crushing was not significant. In 1D compression tests, where applied stresses may exceed the fracture strength of sand grains, grain orientation could influence comminution and compressibility.

**Figure 2:**
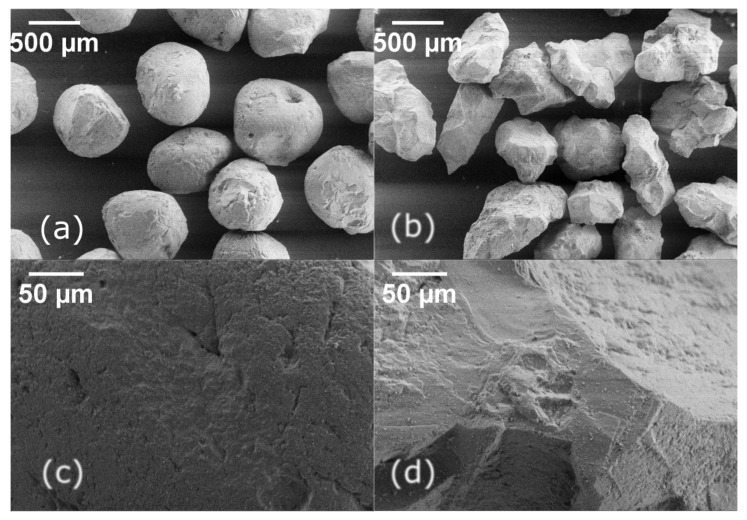
SEM micrographs of (**a**) Ottawa sand grains and (**b**) Q-Rok grain at 94× magnification; Texture of (**c**) Ottawa grain and (**d**) Q-Rok grain at 1000× magnification.

**Figure 3:**
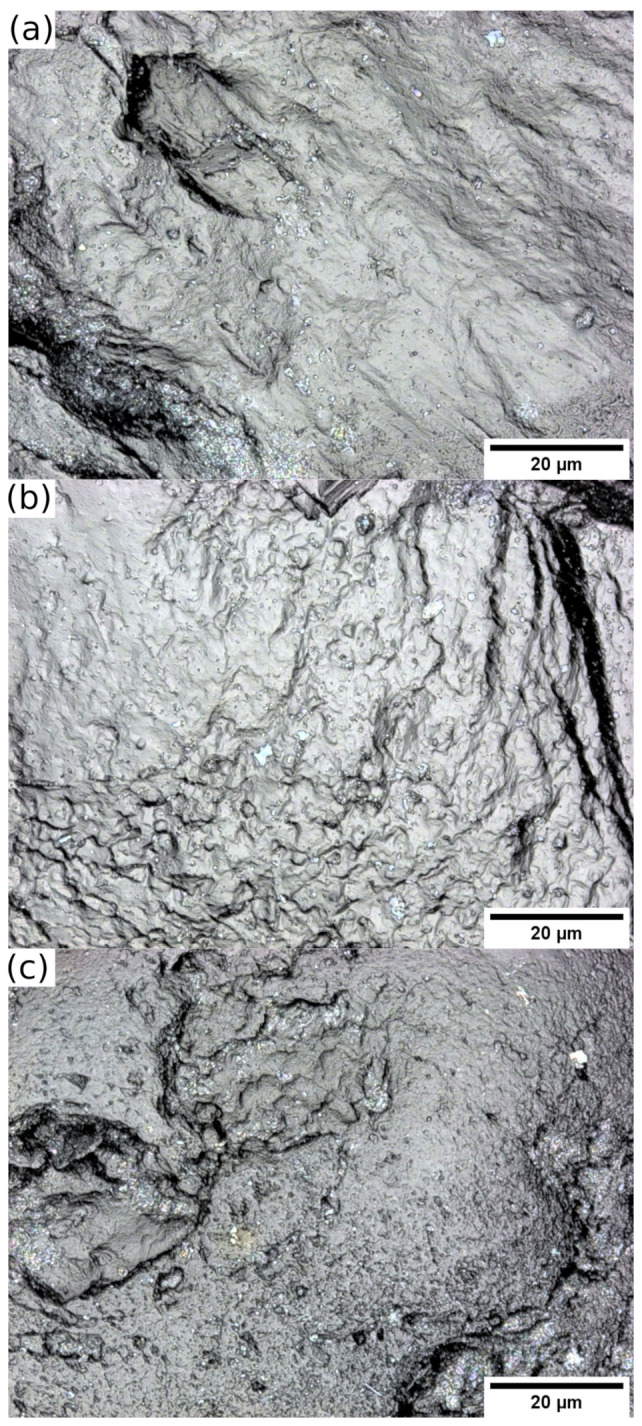
Surface images of (**a**) Ottawa sand, (**b**) Q-Rok, and (**c**) Siligran from high resolution confocal laser microscope.

Scanning Electron Microscope (SEM) images showing the rounded shape of the Ottawa sand and the angular grains of Q-Rok are presented in Figure 2a,b, and high magnification images of the surface of these sands are shown in Figure 2c,d. The defects, depression and holes, on Ottawa sand surface (Figure 2c) and small protrusions on Q-Rok surface (Figure 2d) are a few micrometers in size. The angular ridges on the surface of Q-Rok grains are few hundreds of micrometers contributing to angularity. Prominent angular ridges are absent on Ottawa sand grains. Not visible in the micrographs are multi crystalline nature of larger grains of Q-Rok. Non-contact roughness measurements were made with Keyence VK-X250 confocal laser microscope. Typical surfaces of the three sands are shown in Figure 3. Roughness values are based on 3D surface profiles of six sand grains at 150× magnification and height resolution of at least 12 nm. Surface roughness were computed from 50 μm× 50 μm area after shape correction. Roughness is defined as the mean absolute value of the surface points from the average height. The average roughness values were 0.35, 0.52 and 0.20 μm respectively for Ottawa sand, Q-Rok and Siligran. The roughness values are similar for the three sands though due to angularity the roughness value for Q-Rok is higher than that for Ottawa sand. These values are higher than those reported by [24].

The behavior of coarse-grained material is greatly influenced by its initial state as quantified by relative density. The minimum ($e_{min}$) and maximum ($e_{max}$) void ratios were determined using procedures specified in ASTM Standards [46,47]. Three tests were performed to determine the limiting density for Ottawa sand and Q-Rok and the average values are presented in Table 1. In addition to these ASTM methods, a slight modification of the method presented in [48] was also used to determine the extreme limiting void ratios. Approximately 800 g of sand was poured into a graduated cylinder, 50 g at a time. After each addition the cylinder was lightly tapped with a soft raw-hide mallet six times on four diametrically opposite locations, a total of 24 light taps for $e_{min}$. The volume of the sand was determined to the nearest 10 mL. After minimum void ratio, the top of the cylinder was covered with a stopper, and the cylinder turned upside down and then slowly placed upright again, in 45 s–60 s, to determine $e_{max}$. Three tests were performed to determine the average minimum void ratio, and the average maximum void ratio was determined from ten tests and are presented in Table 1.

The $e_{min}$ and $e_{max}$ using the ASTM method for Ottawa sand were 0.507 and 0.689, and 0.630 and 0.910 respectively for Q-Rok. The $e_{min}$ and $e_{max}$ from the cylinder method were 0.51 and 0.75 respectively for Ottawa sand, 0.60 and 1.01 for Q-Rok, and 0.52 and 0.78 for Siligran. For the same deposition method and energy, the rounded Ottawa sand packs more densely than the angular Q-Rok. Additionally, the difference between the loose and dense state of packing is larger for the angular sand than the rounded sand. The change in void ratio from densification ranged between 32% and 40%, the largest change was in Q-Rok, approximately 40% increase. The values of limiting void ratios from the cylinder method were used when computing the relative densities ($D_{r}$) of the test specimens because similar procedures were adopted for preparing tests specimens. For Ottawa sand, both methods produced similar $e_{min}$, whereas the ASTM method produced a denser packing for the Q-Rok. The angular and multi crystalline nature of Q-Rok, especially the larger grains, may result in some crushing due to vibration of a heavy surcharge in the ASTM method leading to a denser state. Therefore, the cylinder method may be more appropriate for angular sands, and easily crushable materials. Even though the sand is deposited from zero height of drop for $e_{max}$ in the ASTM method, the kinetic energy of the flowing sand particles in vertical drop may result in denser state than in the cylinder method where the sand grains gently roll to rest. The ASTM method may not always produce the densest packing; an air pluviation method with drop height of 40 to 50 cm has been shown to produce a denser state [49]. Siligran is more angular than Ottawa sand, but it also contains more fines. The similarity of index densities of Ottawa sand and Siligran is the result of a combination of the effects of particle size distribution and angularity on the packing density.

## 3. Procedure

Commonly used geotechnical testing methods, such as 1D consolidation, isotropic consolidation, and triaxial testing were adopted to characterize the strength and volumetric response of the specimens. Particle size distribution using sieve analysis, image analysis, and laser diffraction was used to quantify the degree of crushing.

### 3.1. Particle Size Distribution

The particle size distributions were determined using a stack of square mesh openings as per the procedures specified in the Standard Test Methods for Particle Size Distribution (Gradation) of Soils Using Sieve Analysis (D 6913) [50]. In addition to the sieve analysis, laser diffraction technique using a commercially available instrument, Malvern Mastersizer S, was also used to compute the particle size distribution curves. Samples for the laser diffraction were prepared by mixing approximately 1 g of representative mass in 50 ML of water in a glass vial with a cap. The vial was shaken by repeatedly turning it upside down to completely disperse the sand grains in water. A plastic dropper was used to sample from different heights (top, middle and bottom) of the suspension. The steps of shaking the vial and extracting specimen was repeated until enough specimen was gathered for measurement.

Sieve analysis and laser diffraction method do not permit particle shape analysis. Both particle size and shape analysis can however be performed using 2D images of the grains captured at suitable magnification. For proper quantification of the shape and size it is necessary that each grain is represented by adequate number of pixels. The bulk sample were sieved through a 250 μm (No. 60) sieve and the fraction retained on 250 μm was imaged at a lower magnification and the fraction passing 250 μm was imaged at higher magnification. Approximately 1 g of each fraction was then placed in separate vials and mixed in 50 mL of water. The vial was repeatedly shaken by turning it upside down and upon standing, a drop from the top, middle and bottom was placed on the slide. The steps of shaking and sampling were repeated until there was enough specimen on the glass slide without overloading the slide. The slide was then air dried before imaging with optical microscope. The images were analyzed in the image processing software ImageJ [51]. The analysis included conversion to binary image, and separation of the contacting grains using the watershed algorithm. Particle size is reported as the projected surface area’s equivalent diameter.

### 3.2. 1D Compression

The 1D compression specimens were prepared in a steel tube. The internal diameter of the steel tube was 19 mm, and the specimen heights were approximately 20 mm. The loose specimens were prepared by placing sand in the tube from zero drop height. The dense specimens were prepared by compacting the sand in three equal layers. After placing a layer, the tube was tapped on the side five times in four diametrically opposite directions. After tapping, the layer was tamped 25 times. A maximum of 22 kN axial load, equivalent to 77 MPa, was applied to the specimens at the rate of 0.1 mm/min in a displacement-controlled testing system. One-dimensional compression tests are generally performed on specimens with much larger diameter than height to minimize side friction. In the present study the height (H) to diameter (d) ratio was approximately equal to one. The effect of using a smaller H/d ratio specimen is that both vertical and horizontal stresses on the specimen are reduced due to side friction [52], thus resulting in a slightly higher void ratio that would have been achieved with H/d=2.5 specimen.

### 3.3. Isotropic Consolidation

Isotropic consolidation test specimens were prepared in the same manner as the triaxial tests specimens. The confining stress increased in small increments and three cycles of loading and unloading were performed with the maximum effective confining stress of 483 kPa. Each confining stress was held for five minutes before applying the next stress increment.

### 3.4. Triaxial Tests

The triaxial tests were performed on cylindrical sand specimens of 71 mm diameter and 178 mm height. Tests were performed on two relative densities (loose and dense) and for three effective confining stresses of 69 kPa, 103 kPa, and 138 kPa. The loose specimens were prepared by pouring from a slowly rising funnel in a circular pattern maintaining a zero height of drop. The dense specimens were prepared by adding 50 grams of sand to the mold and then lightly tapping the diametrically opposite sides six times with a raw-hide mallet, 24 taps in total for each layer. Carbon dioxide was used to flush air out of the specimens and were subsequently saturated with deaired water and *B* values for all specimens were greater than 0.95. After saturation, the specimens were consolidated at the desired effective stress for one hour. Volumetric strains were calculated by measuring the volume of pore water flowing out of the specimen by differential pressure transducer (DPT). After consolidation, the specimens were sheared at a rate of 12%/hour to a maximum strain of 25%. Data reduction, and calculations including area correction and membrane correction were performed as stated in Standard Test Method for Consolidated Drained Triaxial Compression Test for Soils, (D7181) [53].

## 4. Results and Discussion

The results of the various laboratory tests along with discussions of the results are provided in this section.

### 4.1. Particle Size Distribution

**Figure 4:**
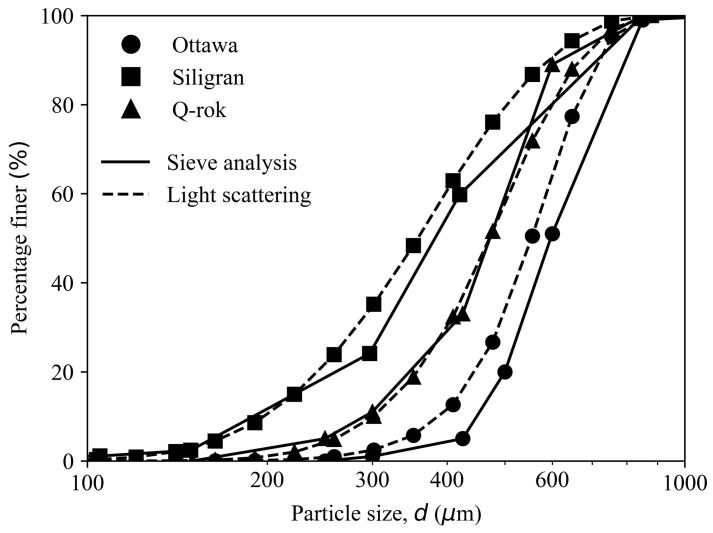
Particle size distribution determined by sieve and laser scattering analysis of Ottawa sand, Q-Rok sand, and Euroquarz Siligran.

The grain size distribution curves from the sieve analysis for the three sands are shown in Figure 4. The maximum particle size for Ottawa and Q-Rok was around 850 μm and the minimum particle size was around 300 μm and 150 μm, respectively. For Siligran the maximum particle size was 710 μm and the minimum particle size was 125 μm. The mean particle size (D50) and coefficient of uniformity (Cu) were 598 μm and 1.43 respectively for Ottawa sand, 470 μm and 1.74 for Q-Rok and 380 μm and 2.21 for Siligran. The classification for all three sands was poorly graded sand (SP) as per the Unified Soil Classification System, ASTM-D2487 [54]. The soil classifications and the values for $D_{50}$, $D_{10}$ and $C_{u}$ are presented in Table 1.

The particle size range for the laser diffraction system was 0.05 μm to 850 μm, thus it was possible to characterize size distribution of uncrushed and crushed specimens using the same instrument. The particle size distributions from sieve analysis are mass-based while those from the laser diffraction are volume distributions. These are equivalent when the specific gravity of the sand particles is constant across the particle size range. The cumulative volume distribution curves for the sands are also shown in Figure 4. The similarity between results of sieve analysis and laser diffraction suggests that with proper sample preparation technique and specimen extraction method, small specimen volumes may not produce large errors.

**Figure 5:**
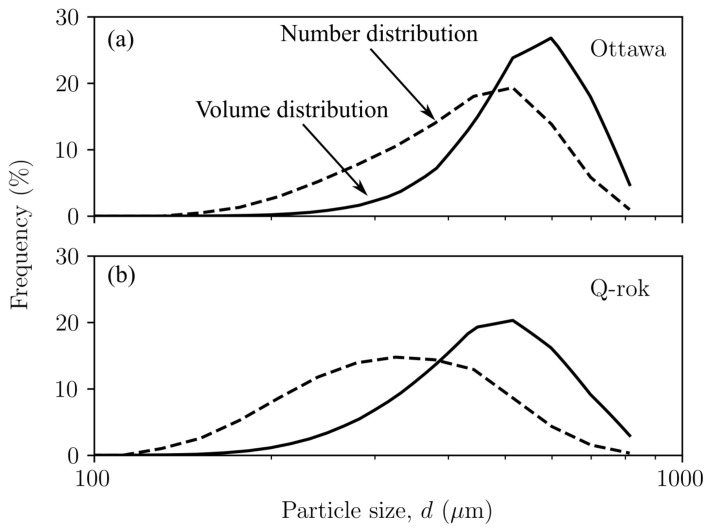
Volume and calculated number distribution of (**a**) Ottawa sand, and (**b**) Q-Rok from laser light scattering technique.

The volume distribution can be converted to number distribution of equivalent sphere sizes albeit with the introduction of certain error due to the assumption of spherical particle shape. The volume distribution and number distribution obtained from the light scattering technique for uncrushed Ottawa sand and Q-Rok are shown in Figure 5. The $D_{50}$ of the volume distribution for Ottawa sand and Q-Rok is 514 mm and 435 mm respectively, and 405 mm and 302 mm in the number distribution. The difference in volume and number distribution is greater in Q-Rok because of the presence of finer particles.

**Figure 6:**
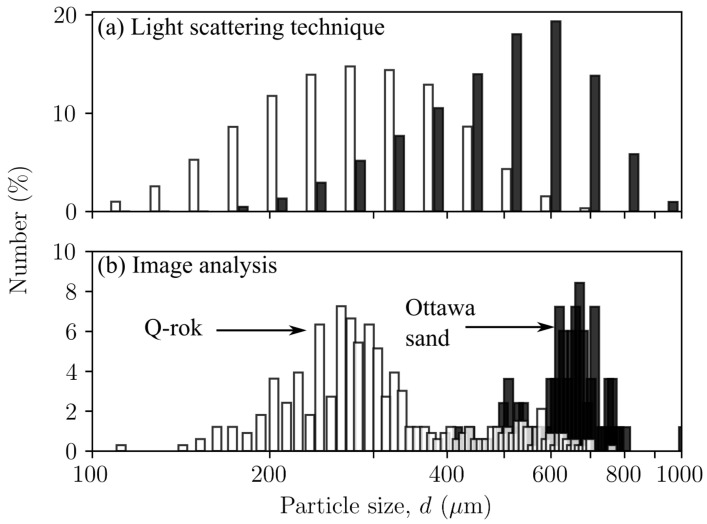
Number distribution from (**a**) laser light scattering technique, and (**b**) image analysis for Ottawa sand and Q-Rok.

Image analysis produces a number distribution in which the number of particles for a given size range is counted from 2D images. The number-based particle size distribution from the laser diffraction technique and the image analysis for the Ottawa sand and Q-Rok are shown in Figure 6. The distribution is based on 83 Ottawa sand grains and 331 Q-Rok particles. The size distributions from both the methods are similar. A major advantage of image analysis over sieve analysis and laser light scattering technique is that it can be used for shape analysis for determining the evolution of grain shape in particle crushing.

The image analysis evaluates particle sizes as equivalent area diameter based on 2D images. This method also suffers from small specimen volume. Less than 1 mg of sand was deposited on the glass slides for imaging. However, with proper sampling technique the results from image analysis are not very different from those obtained from the laser light diffraction technique for number distribution as shown in Figure 6. Though, it is not a common practice, particle size distributions from image analysis and laser diffraction have even been combined to study particle crushing [37].

### 4.2. 1D Compression

**Figure 7:**
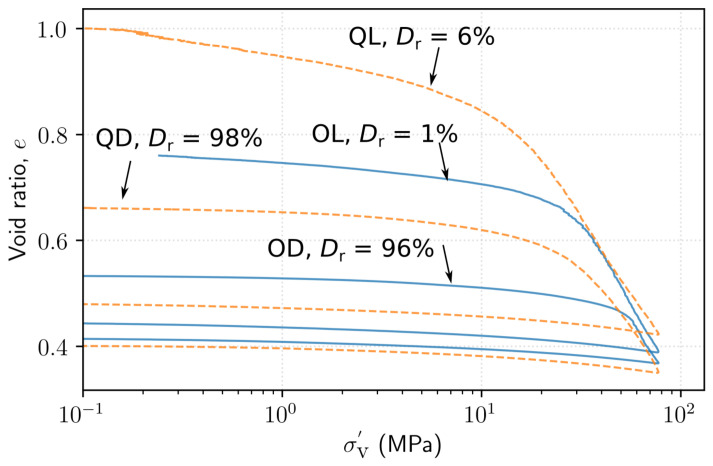
Results of displacement-controlled 1D compression tests on loose (L) and dense (D) specimens of Ottawa sand (O) and Q-Rok (Q).

The results of the 1D compression tests for the loose and dense specimens of Ottawa sand (OL and OD) and Q-Rok (QL and QD) are presented in Figure 7. The initial void ratios for OL and OD were 0.76 and 0.53 respectively while those for QL and QD were 1.01 and 0.67. Threshold stress is defined as the stress where the slope of the curve increases appreciably with the initiation of particle crushing. The threshold stresses for OL and OD were 27 MPa and 48 MPa respectively, and 12 MPa and 22 MPa for QL and QD, on average an 80% increase. Densification increases the threshold stress and the threshold stress for QD was comparable to OL but smaller than OL. The response of QD was similar to OL in terms of threshold stress, void ratio reduction and compression index. At high stresses the normal compression lines (NCL) for OL, OD and QD converge and approach the same compression index ($C_{c}$) value of 0.60 at around 70 MPa axial stress. The $C_{c}$ for QL was 0.60 until 50 MPa after which the curve starts to flatten resulting in a smaller value of 0.55. No effort was made to reduce the side wall friction and hence the void ratios in these tests may be higher than they would be if side walls were frictionless [52]. The frictional sidewall and H/d=1 may also explain the higher final void ratio for loose specimens than dense specimens as loose specimens experience larger strain, and hence more energy may be lost in sidewall friction. At high stresses beyond the threshold stress, NCLs for specimens prepared at different densities converge, thus resulting in a unique NCL [27,52]. Though the grain shapes are very different, the grading of Ottawa sand and Q-Rok are similar, which may have led to the convergence of NCL. At high enough stresses particle breakage negates the influence of particle shape for similarly graded specimens to approach a unique NCL. The void ratios at the maximum stress were 0.388, 0.367, 0.423 and 0.350 for OL, OD, QL and QD respectively.

In terms of axial strain, the axial strain at the maximum axial stress of 77 MPa for the OL and OD were 21% and 11% respectively and those for QL and QD were 29% and 19%. On average, the improvement in stiffness is around 40%. The loose specimens compressed significantly more than the dense specimens; 10% more axial strain. The axial strain in Q-Rok specimens are 8% larger than those for the Ottawa sand specimens for similar packing density. The elastic strain recovery for all specimens was around 3%.

**Figure 8:**
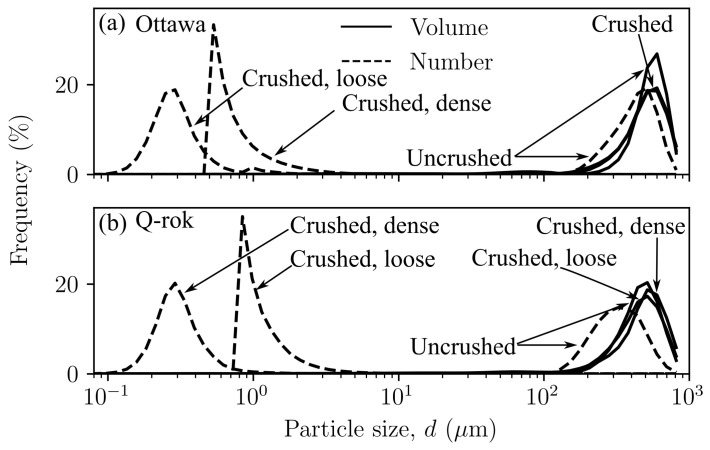
Particle size evolution in dense and loose specimens of (**a**) Ottawa sand and (**b**) Q-Rok subjected to 1D compression loading, shown as volume and number distributions.

The particle size distribution before and after 1D compression tests in terms of volume and number distributions are shown in Figure 8. All crushed specimens were subjected to a maximum axial stress of 77 MPa in 1D compression. There are very small differences in the volume distributions of uncrushed (as received) sands and crushed sands after 1D compression. This is possibly due to small number of larger particles dominating the distribution in the crushed samples after 1D compression. Considerable differences are highlighted by the number distributions. There is significant increase in the number of fines in the micrometer and sub-micrometer size range. Using Hertzian contact, surface flaws, simple linear fracture mechanics, and with the assumption of linear scaling of flaw size to particle size, Zhang et al. [55] calculated flaw sizes based on threshold stress in 1D compression tests. Their values for flaw sizes in quartz ranged from 0.004 to 0.07 μm. Brzesowsky et al. [56] using similar approach computed equivalent values from single grain crushing tests. The flaw size reduces with particles size and Kanda et al. [57] and King and Bourgeois [58] have reported increasing higher energies are required to crush smaller particles. This may lead to a comminution particle size limit which is orders of magnitude larger than the flaw size. Kendall [59] estimated this limit to be 1 μm. However, particle size analysis of crushed particles in laboratory projectile penetration tests indicate that sands can be crushed to sub-micron particle size [42].

Visualizing particle size distribution in both mass/volume and number distribution provides a more complete information in understanding the role of coordination number in comminution. Since the average force experienced by a grain depends on the number of contacting grains [60], the number distribution could provide useful insights into the comminution process. The nature of ultimate crushing is fractal and may be uniquely determined by the maximum particle size before crushing [31,40,61]. It is generally agreed that crushing begins from smaller particles as they have fewer number of contacting neighbors and smaller coordination number [62]. As the smaller grains around larger grains fracture, either confining the larger particles or filling up the voids, increasing the coordination number for the larger particles, it becomes increasingly difficult to fracture these larger grains. Thus, smaller particles with smaller coordination number continue to crush [63]. Though, in general larger grains have higher coordination number [64] they also experience larger forces [64]. In addition, specific energy (energy per unit mass) required to fracture single grains increases with decreasing particle size [57,65]. Thus, a significantly larger energy is required to fracture smaller particles. Particle crushing has been observed to begin from larger grains [55]. In view of the above observations, especially in poorly graded (containing very few different-sized grains) assemblies particle crushing is more likely to begin from larger grains. The crushing of larger grains continue until enough fines have been produced to surround the larger grains to offset the lower energy required to crush larger grains. This is evident, from the number distribution, as the number of small particles has increased significantly, and at the same time the volume distribution still indicates the presence of a few larger particles.

Particle crushing is generally quantified using relative breakage, $B_{r}$. This is generally computed as the ratio of the area between before and after crushing PSD curves and the area above the before crushing PSD, with 74 μm as the lower limit of the PSD [32]. This assumes that at ultimate crushing all the particles are finer than 74 μm. Einav [31] modified Hardin’s breakage factor by assuming a fractal nature for the ultimate distribution as given by Equation (1):

(1) $$F_{u}\left(d\right)={\left(d_{m}/d_{M}\right)}^{3-\alpha }$$

where $Fu\left(d\right)$ is the ultimate cumulative PSD, *d* is the particle size in the units of length, $d_{m}$ and $d_{M}$ are the minimum and maximum particle sizes, α is the fractal dimension taken to be 2.6 [31,66], $F\left(d\right)$ is the current cumulative PSD, and $F_{0}\left(d\right)$ is the initial cumulative PSD. The crushing in 1D compression is quantified using as Equation (2) [31].

(2) $$B_{r}=\frac{\int_{d_{m}}^{d_{M}}\left(F\left(d\right)-F_{0}\left(d\right)\right)dd}{\int_{d_{m}}^{d_{M}}\left(F_{u}\left(d\right)-F_{0}\left(d\right)\right)dd}$$

The value of $B_{r}$ ranges from 0 for no crushing to 1 for complete crushing. The values of $B_{r}$ for OL and OD were 0.12 and 0.15 and 0.10 and 0.09 for QL and QD respectively, with values for Ottawa sand higher than Q-Rok as shown in Table 2. Additionally presented are relative crushing values as per Lee and Farhoomand [29]. They defined relative crushing ($B_{r}$) as the ratio of $D_{15}$, particle size at which 15% of the material was finer, before and after crushing (D_15i_/D_15a_). The $D_{15}$ of the crushed specimens, from the volume distribution, is smaller by 100 μm in Ottawa sand and by 50 μm in Q-Rok irrespective of the density. The relative crushing values are similar for both loose and dense packing. The extent of crushing is more evident in the number distribution in which the D15 values are smaller than 1 μm, with 200–1000 times more particles than the uncrushed specimen. In general, soils with angular grains show more particle crushing than rounded grains [29]; however, the small differences in the PSD of Ottawa sand and Q-Rok may have contributed to more crushing in Ottawa sand specimens than in Q-Rok specimens.

**Figure 9:**
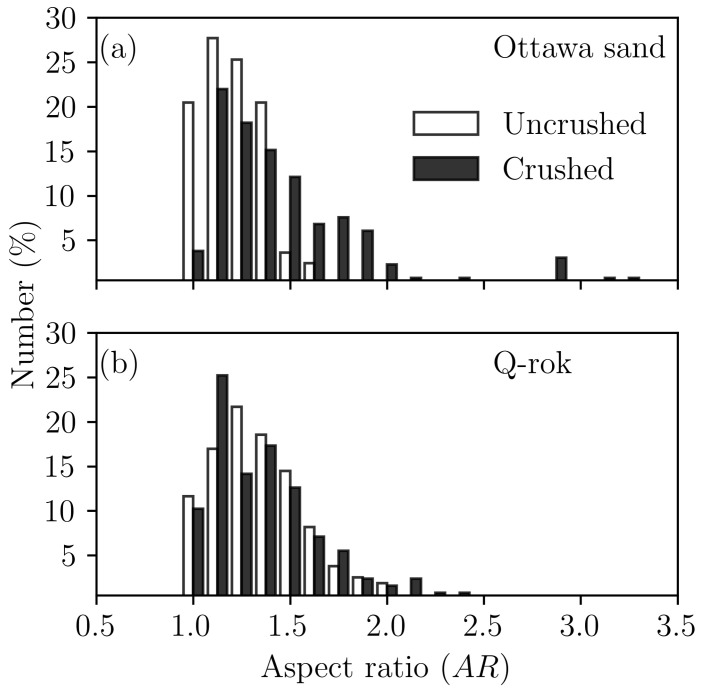
Particle shape evolution after 1D compression loading: (**a**) Ottawa sand, (**b**) Q-Rok.

The evolutions of particle shape during 1D compression for OD and QD are shown in Figure 9. There is an increase in the aspect ratio of the particles for OD and decrease in the aspect ratio of the particles for QD. Aspect ratio is defined as the ratio of the major to the minor axis of the ellipse fitted to the 2-D image of the particles. The implication is that rounded particles tend to fracture diametrically or cordially thus increasing the aspect ratio of the crushed particles [67], while asperities breaking in angular particles tend to make angular particles more rounded. The aspect ratio may continue to increase with increasing compressive stress [27].

### 4.3. Isotropic Consolidation

**Figure 10:**
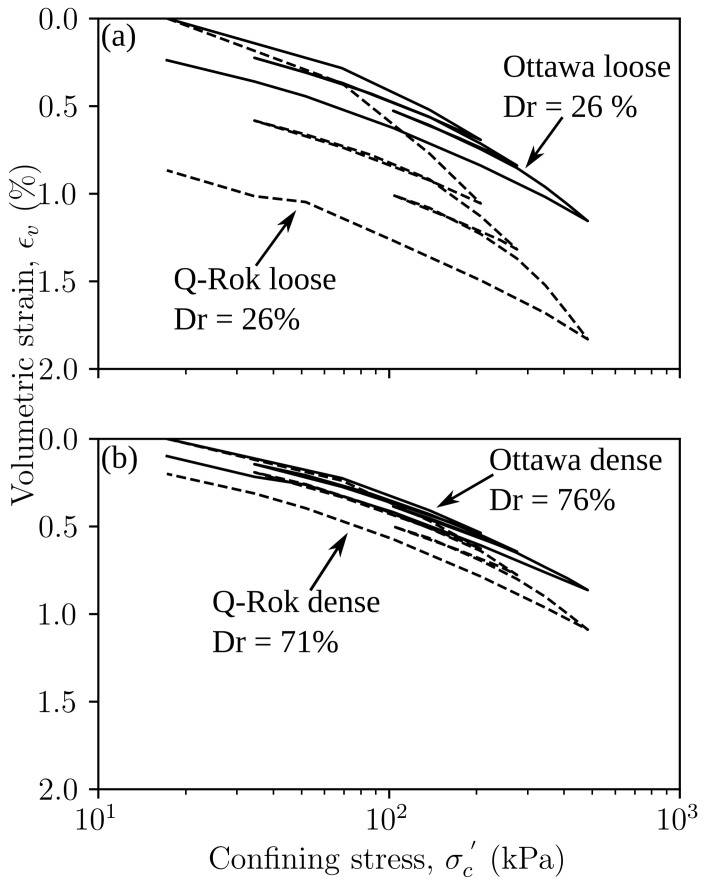
Isotropic consolidation of (**a**) loose, and (**b**) dense specimens of Ottawa sand and Q-Rok.

The results for the isotropic consolidation tests performed on loose and dense specimens of Ottawa sand and Q-Rok are shown in Figure 10. The volumetric response is non-linear over the range of stress applied. For the Ottawa sand, the volumetric strains at the maximum confining stress, $\sigma c^{\prime }$, of 483 kPa was 1.16% for the loose specimen and 0.86% for the dense specimen. For the Q-Rok specimens the volumetric strains were 1.83% and 1.09% for the loose and dense specimens, respectively. The elastic rebound upon unloading to 17.2 kPa ranged from 0.92% to 0.76% for loose and dense specimens of Ottawa and 0.96% to 0.89% for loose and dense specimens of Q-Rok. There is significant improvement in volumetric behavior from densification. The total volumetric strain for the dense Q-Rok is similar to that for the loose Ottawa sand. The volumetric strain is influenced by both relative density and particle shape. However, particle morphology has a greater influence at low relative density. In addition, the total volumetric strain of the loose Ottawa sand specimen with $e_{0}$ of 0.69 and the dense Q-Rok specimen with $e_{0}$ of 0.72 are similar. This observation along with the 1D compression results indicate that void ratio could be a bigger influence than particle morphology in volumetric response under 1D and isotropic compression.

### 4.4. Triaxial Tests

**Figure 11:**
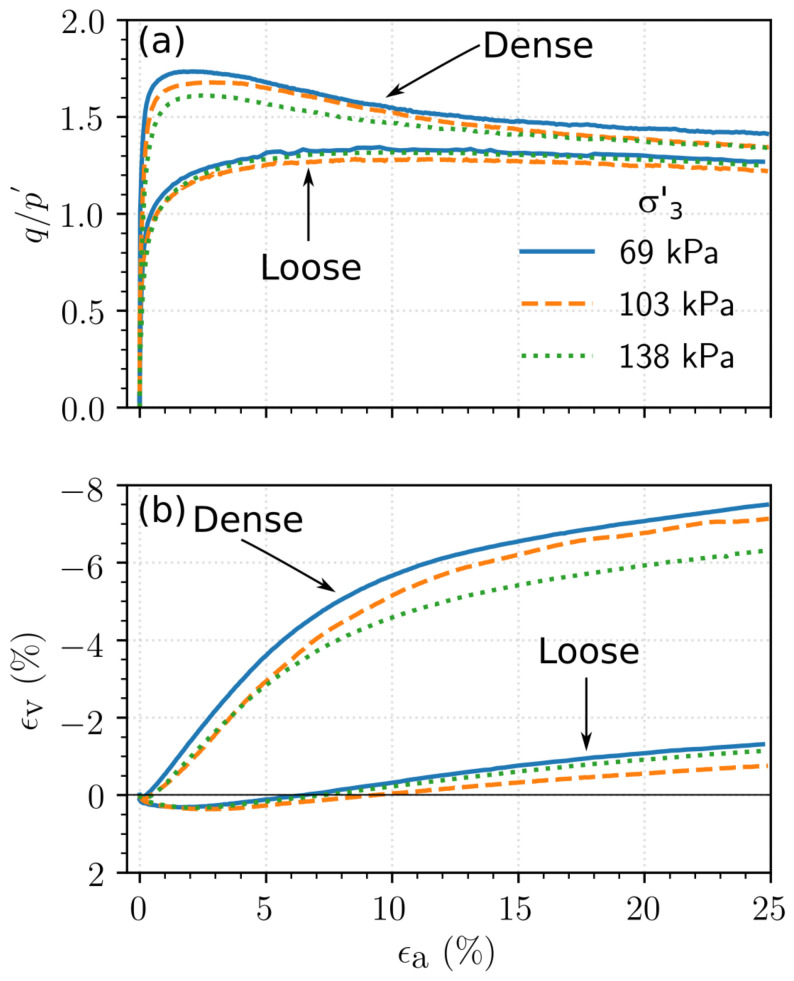
Response of Ottawa sand to drained triaxial compression: (**a**) Stress–strain relationship, (**b**) Volumetric relationship.

**Figure 12:**
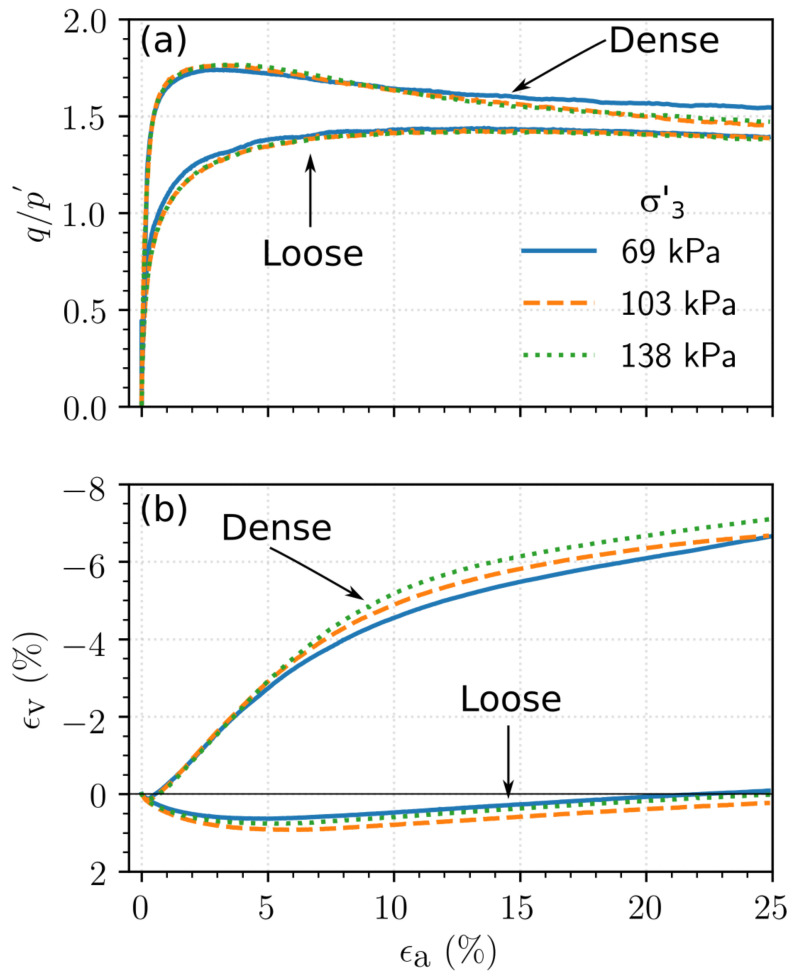
Response of Q-Rok to drained triaxial compression: (**a**) Stress–strain relationship, (**b**) Volumetric relationship.

**Figure 13:**
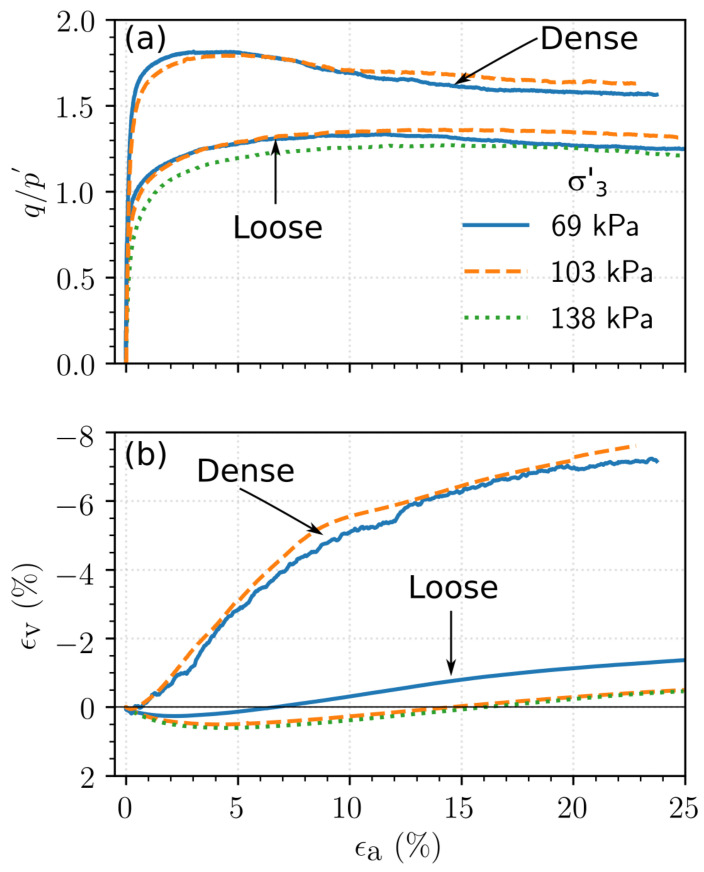
Response of Euroquarz Siligran to drained triaxial compression: (**a**) Stress–strain relationship, (**b**) Volumetric relationship.

The results of the consolidated drained triaxial tests performed on the loose and dense specimens of Ottawa sand, Q-Rok and Siligran are shown in Figure 11, Figure 12 and Figure 13 respectively. The initial specimen states and pertinent test results are presented in Table 3. If no peak stress was observed, failure strain, $\epsilon_{p}$, was chosen to be 15%. The stress ratio is defined as the ratio of the deviatoric stress and mean effective stress, $q/p^{\prime }$, in which *p* and *q* are computed as shown Equation (3):

(3a) $$\begin{array}{c} p^{\prime }=\left(2\sigma_{c}^{\prime }+\sigma_{a}^{\prime }\right)/3 \end{array}$$

(3b) $$\begin{array}{c} q=\sigma_{a}^{\prime }-\sigma_{c}^{\prime } \end{array}$$

where $\sigma_{c}^{\prime }$ and $\sigma_{a}^{\prime }$ are axial and confining stresses in a triaxial test and are equivalent to major and minor principal stresses.

**Figure 14:**
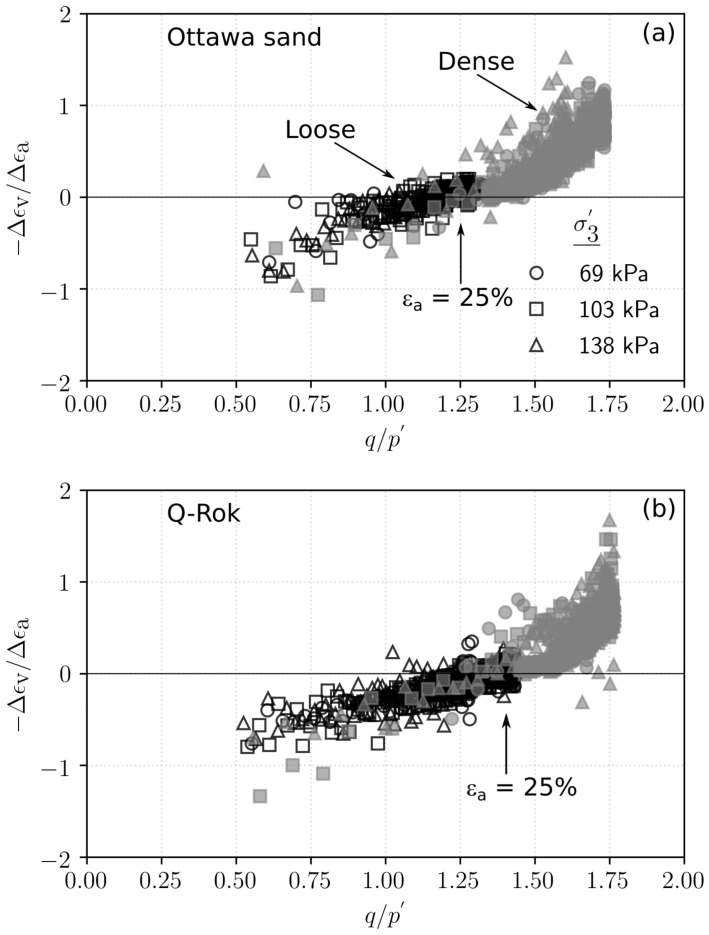
Stress–dilatancy plot showing the current rate of dilation for (**a**) dense and loose specimens of Ottawa sand, (**b**) dense and loose specimens of Q-Rok.

**Figure 15:**
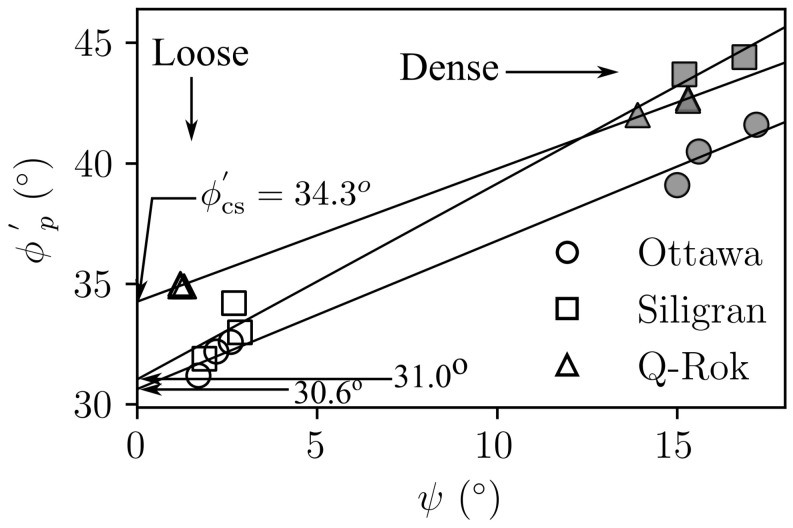
Determination of critical state friction angle.

The stress ratio, $q/p^{\prime }$, for dense specimens ranged from 1.68 for Ottawa sand to 1.81 for Siligran. At axial strain, $\epsilon_{a}=25\%$, $q/p^{\prime }$ was 1.25 for the loose specimens of Ottawa sand and Siligran and 1.4 for Q-Rok. All specimens continued to dilate even at large strains possibly due to formation of new shearing bands as seen in 3D computed tomography images [68,69,70]. Dilation was considerably greater for the dense specimens than for the loose specimens. The current rate of dilation with respect to $q/p^{\prime }$ is shown in Figure 14 for loose and dense specimen of Ottawa sand and Q-Rok. The loose specimens show very little dilation throughout the duration of the tests. The maximum dilation rate occurs at peak $q/p^{\prime }$ and is higher for Q-Rok than for Ottawa. It decreases with increasing $q/p^{\prime }$ after the peak. The stress ratios for loose specimens at 25% axial strain are marked on the figures. The $q/p^{\prime }$ at $\epsilon_{a}=25\%$ for dense specimens were 1.36 for Ottawa sand and 1.48 for Q-Rok. The dilation rates for these $q/p^{\prime }$ are also approaching zero though they are not close to zero as in the loose specimens. Even at large strains dilation rate for dense specimens are considerable. Frictional end triaxial tests suffer from strain localization which may start early during the shearing stage depending on the density and confining stress [71]. Localized volumetric strains in regions of active deformation are different from global volumetric strains [72] which are measured from the volume of pore water flowing in or out of the specimen. These global volume strains are then used to correct axial stresses by assuming uniform deformation. Hence, stress values and by extension critical state friction angle, $\phi_{cs}^{\prime }$, computed based on these global volume strains may not reflect the true value. Bolton [73] proposed a simple saw blade model in which the peak friction angle, $\phi_{p}^{\prime }$, is the sum of $\phi_{cs}^{\prime }$ and some fraction, *k*, of the dilation angle, ψ, $\left(\phi_{p}^{\prime }=\phi_{cs}^{\prime }\;+k\psi \right)$. Bolton proposed k=0.8 for plane shear and approximately 0.5 for triaxial shear. Guo and Su [22] have reported *k* values of 0.63 for Ottawa sand and 0.91 for angular crushed limestone. The dilation angle was determined using the equation proposed by Vermeer and de Borst [74]. In these series of tests, *k* for angular grains ranged from 0.55 for Q-Rok, to 0.81 for Siligran. The value for Ottawa sand was 0.62. The value of *k* appears sensitive to void ratio and angularity. Q-Rok which is more angular but has a lower density than Siligran has a smaller *k* value. However, Siligran which is more angular than Ottawa sand but has similar density shows higher *k* value. Critical state friction angles determined using Bolton’s model is shown in Figure 15. The value for Ottawa sand is smaller than that for Q-Rok, displaying the influence of particle shape on $\phi_{cs}^{\prime }$. A similar approach, using rate of dilation, was used by Vaid and Sasitharan [75] to determine $\phi_{cs}^{\prime }$ for Erksak sand with excellent agreement.

**Figure 16:**
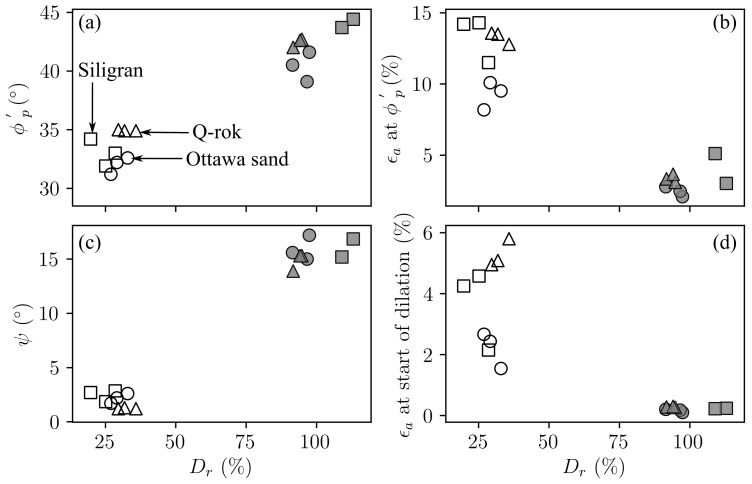
Effect of $D_{r}$ on strength and volume parameters in conventional drained triaxial testing: (**a**) peak friction angle, (**b**) axial strain at peak friction angle, (**c**) dilation angle, (**d**) axial strain at the start of dilation.

There were two major variables in this suite of tests: density and particle morphology. The effect of densification on test parameters are shown in Figure 16. With increasing $D_{r}$, both the $\phi_{p}^{\prime }$ and ψ increases approximately by the same amount suggesting that the strength gain due to densification, increase in $\phi_{p}^{\prime }$, is primarily from dilation. The peak friction angle was defined at maximum deviatoric stress, and the point of maximum compression was considered to be the start of dilation.

**Figure 17:**
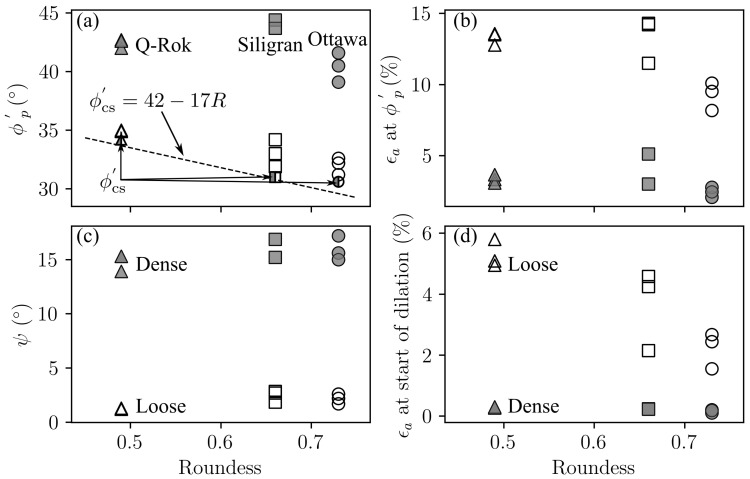
Effect of particle shape on strength and volume parameters in conventional drained triaxial testing: (**a**) peak friction angle, and the critical state friction angle relationship [12], (**b**) axial strain at peak friction angle, (**c**) dilation angle, (**d**) axial strain at the start of dilation.

The effect of particle shape on test parameters are shown in Figure 17. Particle interlocking in angular sands leads to higher friction angle [22] as shown in Figure 17a. Though, Siligran is less angular than Q-Rok, the specimens are relatively denser thus the higher $\phi_{p}^{\prime }$ for the dense Siligran specimens. Additionally shown in Figure 17a are $\phi_{cs}^{\prime }$ from Figure 15 and the relationship between roundness (*R*) and $\phi_{cs}^{\prime }$ [12]. From Figure 16 and Figure 17, in general, relative density has a significantly greater influence on the behavior of granular specimens than angularity. However, the influence of angularity on $\phi_{p}^{\prime }$ is greater in loose specimens than in dense specimens. This is because angularity and the associated roughness affects the critical state behavior, $\phi_{cs}^{\prime }$, as shown in Figure 17a. However, the increase in strength from densification, ψ, is similar for the different sands as seen in Figure 17c.

## 5. Conclusions

One-dimensional compression of rounded and angular sands at loose and dense packing confirm the existence of a unique NCL line for similar grading irrespective of particle shape. Analysis of comminuted sand after 1D compression reveal that rounded sands become more angular while angular sands become more rounded. Particle size distribution of crushed specimens from laser diffraction showed presence of sub-micron fines after crushing, for both rounded and angular specimens. In addition, both volume and number can be used to better understand the comminution process. Presence of many fines and a few large particles indicate particle crushing starts in larger particles. The role of density and particle shape on strength and volume parameters in drained triaxial compression tests on natural sands with similar composition, grain size distribution and surface roughness but very different particle shape were presented. Relative density has a greater influence than particle shape on strength and volumetric parameters. Particle morphology exerts a greater influence in loose specimens than in dense specimens. Densification of angular material may provide the benefit of higher friction angle and the stiffness, approximately 40% increase, equivalent to that of rounded material.

A further extension of this study would be to image particle crushing under 1D compression using X-ray microtomography.

## Figures and Tables

**Table 1:** Void ratio, and some index properties for Siligran 0.125–0.71, Ottawa sand, and Q-Rok.

Parameter	Siligran 0.125 to 0.71	Ottawa Sand	Q-Rok
ASTM method			
$e_{\min}$		0.507	0.630
$e_{\max}$		0.689	0.901
Cylinder method			
$e_{\min}$	0.52	0.51	0.60
$e_{\max}$	0.78	0.75	1.01
Grain size distribution			
$d_{50}$ [m]	380	595	475
$d_{10}$ [m]	190	465	300
$C_{u}$	2.21	1.37	1.67
Classification	SP	SP	SP

**Table 2:** Breakage values computed using volume and number distribution using Lee and Farhoomand [29] equation and volume distribution using Einav [31] ultimate fractal distribution method.

Specimen	Volume [29]			Number [29]			Volume [31]
	D15i	D15a	Br	D15i	D15a	Br	Br
OL	389.9	288.1	1.35	266.0	0.19	1401	0.12
OD	389.9	283.1	1.38	266.0	0.53	502	0.15
QL	305.6	257.0	1.19	198.0	0.84	235	0.10
QD	305.6	269.0	1.14	198.0	0.20	988	0.09

**Table 3:** Initial states, and results of consolidated drained triaxial compression of Ottawa sand, Q-Rok, and Siligran.

Specimen	Dr [%]	$e_{0}$	$e_{c}$	$\sigma_{c}^{\prime }$ [kPa]	$\phi_{p}^{\prime }$ [${}^{\circ }$]	$\epsilon_{f}$ [%]	ψ [${}^{\circ }$]
OL1	32.9	0.669	0.664	69	32.6	9.5	3.4
OL2	26.9	0.683	0.676	103	31.2	8.2	2.2
OL3	29.1	0.678	0.669	138	32.2	10.1	2.9
OD1	97.4	0.518	0.515	69	41.6	2.1	22.2
OD2	91.5	0.532	0.527	103	40.5	2.8	21.1
OD3	96.6	0.520	0.514	138	39.1	2.5	23.6
QL1	29.6	0.917	0.907	69	35.0	13.6	1.5
QL2	35.8	0.897	0.885	103	34.9	12.8	1.7
QL3	31.8	0.910	0.898	138	34.9	13.5	1.6
QD1	91.7	0.716	0.711	69	42.0	2.6	14.8
QD2	94.8	0.706	0.700	103	42.7	3.1	15.6
QD3	94.1	0.708	0.700	138	42.6	3.1	15.1
SL1	28.5	0.706	0.701	69	33.0	11.5	2.85
SL2	19.7	0.728	0.721	103	34.2	14.2	2.69
SL3	25.1	0.714	0.704	138	31.9	14.3	1.86
SD1	113	0.485	0.482	69	44.4	3.0	16.7
SD2	109	0.495	0.491	103	43.7	5.1	15.2

OL = Ottawa loose, OD = Ottawa dense, QL = Q-Rok loose, QD = Q-Rok dense, *D*r = Relative density, $e_{0}$ = Initial void ratio, $e_{c}$ = Void ratio after consolidation, $\sigma_{p}^{\prime }$ = Consolidation stress, $\phi_{p}^{\prime }$ = Peak friction angle, $\epsilon_{p}$ = Axial strain at peak friction angle, ${}^{\psi }$ = Dilation angle.

## Data Availability

The authors Will provide data upon request.
